# Concentration of Lactoferrin in Human Milk and Its Variation during Lactation in Different Chinese Populations

**DOI:** 10.3390/nu10091235

**Published:** 2018-09-05

**Authors:** Zhenyu Yang, Rulan Jiang, Qi Chen, Jie Wang, Yifan Duan, Xuehong Pang, Shan Jiang, Ye Bi, Huanmei Zhang, Bo Lönnerdal, Jianqiang Lai, Shian Yin

**Affiliations:** 1 National Institute for Nutrition and Health, Chinese Center for Disease Control and Prevention, Beijing 100050, China; yangzy@ninh.chinacdc.cn (Z.Y.); wangjie@ninh.chinacdc.cn (J.W.); duanyf@ninh.chinacdc.cn (Y.D.); pangxh@ninh.chinacdc.cn (X.P.); jiangshan@ninh.chinacdc.cn (S.J.); biye@ninh.chinacdc.cn (Y.B.); zhanghm@ninh.chinacdc.cn (H.Z.); shianyin@126.com (S.Y.); 2 Department of Nutrition, University of California, Davis, CA 95616, USA; rjiang@ucdavis.edu (R.J.); bllonnerdal@ucdavis.edu (B.L.); 3 Zhejiang Center for Disease Control and Prevention, Hangzhou 310000, China; chenqi1982@163.com

**Keywords:** Lactoferrin, concentration, breast milk, maternal factors, Chinese populations

## Abstract

Background: Lactoferrin (Lf) is a multifunctional protein and one of the most abundant proteins in human milk. Various factors may affect its concentration in human milk, such as stage of lactation, ethnicity, and diet. Objectives: The objectives of the present study were to examine the dynamic change in milk Lf throughout the course of lactation and explore factors associated with milk Lf concentrations in various Chinese populations. Methods: This investigation was a part of a large cross-sectional study conducted in 11 provinces/autonomous regions/municipalities (Beijing, Gansu, Guangdong, Guangxi, Heilongjiang, Inner Mongolia, Shandong, Shanghai, Xinjiang, Yunnan, and Zhejiang) across China between 2011 and 2013. Lactating women (*n* = 6481) within 0–330 days postpartum were recruited in the original study. A sub-sample of 824 women was randomly selected, and milk Lf concentrations were determined by UPLC/MS. Results: The Lf concentration in milk from women delivering at term was 3.16 g/L, 1.73 g/L and 0.90 g/L for colostrum, transitional milk, and mature milk, respectively. Lf concentrations differed significantly between stages of lactation (colostrum vs. transitional milk, colostrum vs. mature milk, transitional milk vs. mature milk, all *p* < 0.001). Maternal BMI, age, mode of delivery, parturition, protein intake, and serum albumin concentration were not correlated with milk Lf concentration. However, milk Lf concentrations varied among different geographical regions (Guangdong (1.91 g/L) vs. Heilongjiang (1.44 g/L), *p* = 0.037; Guangdong (1.91 g/L) vs. Gansu (1.43 g/L), *p* = 0.041) and ethnicities (Dai (1.80 g/L) vs. Tibetan (0.99 g/L), *p* = 0.007; Han (1.62 g/L) vs. Tibetan (0.99 g/L), *p* = 0.002) in China. Conclusions: The concentration of Lf in human milk changes dynamically throughout lactation. Few maternal characteristics affect the milk Lf concentration, but it varies across different geographical regions and ethnicities in China.

## 1. Introduction

Lactoferrin (Lf) is an iron-binding glycoprotein involved in a wide range of biological functions including anti-infectious, antimicrobial, anti-inflammatory, immunomodulatory, and prebiotic activities, as well as promotion of cellular proliferation and differentiation [1,2]. Importantly, clinical trials have demonstrated roles for Lf in prevention of diarrhea [3,4], neonatal sepsis, and necrotizing enterocolitis in preterm infants [5,6,7]. It is secreted by exocrine glands and secondary neutrophils and mainly present in external secretions such as milk, saliva, tears, bile, and pancreatic fluids [8]. Lf is particularly abundant in human colostrum (~7 g/L) and mature milk (~1 g/L), comprising 15–20% of the total protein content [9]. Compared to human milk, cow milk has a relatively low Lf concentration: 1.5 mg/L in colostrum and 0.5 mg/L in mature milk [10]. As the second most abundant protein and a multifunctional protein in human milk, Lf may significantly contribute to better health, growth and development of infants and later outcomes, which are associated with breastfeeding. Thus, it is important to understand factors affecting the intake of Lf during infancy.

Recent literature has shown that concentrations of Lf in milk vary widely [11,12,13]. Both maternal and infant factors may affect the concentration of Lf in human milk. Milk Lf concentrations are consistently highest in colostrum and then decrease gradually. However, it is unclear whether geographic location, ethnicity, maternal age, parity, socioeconomic status, nutritional status, infant infections, prematurity, and type of delivery are associated with the concentration of Lf in breast milk.

Changes in concentration of Lf in milk throughout the course of lactation have not been well documented in Chinese populations. The objectives of the present study were to examine the dynamic changes in milk Lf concentrations during lactation and to explore maternal and infant factors associated with milk Lf concentrations in different Chinese populations.

## 2. Methods

### 2.1. Study Design

This study was part of a large study on Chinese human milk composition. The detailed study information was reported previously [14]. Briefly, this was a cross-sectional study conducted in 11 provinces/autonomous regions/municipalities (Beijing, Gansu, Guangdong, Guangxi, Heilongjiang, Inner Mongolia, Shandong, Shanghai, Xinjiang, Yunnan, and Zhejiang) across China between 2011 and 2013. The study sites were located in northern, southern, western, and eastern China. Both inland areas and coastal areas were covered in the study.

### 2.2. Subjects

Lactating women within 0–330 days postpartum and their infants were recruited in the study. Besides Han ethnicity, minor ethnicities (Bai, Dai, Hui, Mongolian, Tibetan, Uygur, and Zhuang) were included. In total, 6481 dyads of self-reported healthy lactating mothers and their infants were recruited in the original study. A sub-sample of 824 was randomly selected from the original study according to lactation stage. A structured questionnaire was used to collect socio-economic status, demographic status, life style, gestational and parturition information, birth outcome, breastfeeding information, and introduction of complementary foods, which were described previously [14]. A food frequency questionnaire was used to investigate maternal food consumption one month prior to milk collection. This study was approved by the Medical Ethics Committee of the National Institute for Nutrition and Food Safety, Chinese Center for Disease Control and Prevention (CCDC). Signed consent was obtained from all subjects.

### 2.3. Human Milk Processing

Human milk was collected using a portable electronic breast pump (HNR/X-2108Z, Shantou, Guangdong, China) in the morning (9 a.m. to 11 a.m.). A complete expression from one breast was pumped into a feeding bottle. The sample was then aliquoted into 15 mL centrifuge tubes and stored in a −20 °C freezer in the field. Samples were subsequently transported to a central lab at the National Institute for Nutrition and Health, Chinese Center for Disease Control and Prevention in Beijing and stored in a −80 °C freezer until analysis.

### 2.4. Lf Measurement

Human milk Lf was measured using ultra-high performance liquid chromatography-tandem (UPLC)/mass spectrometry (MS) with isotopic dilution. Details of the method have been published previously [15]. In brief, human milk proteins were hydrolyzed using trypsin, and the supernatant was analyzed using UPLC/MS. A signature peptide VPSHAVVAR of human Lf (amino acid residues of 269–277) was identified, and the isotope-labelled signature peptide, VPSHAV*V*AR (V*, Val-OH-^13^C5, ^15^N), was used as an internal standard for quantification. Samples and standards were separated with the ACQUITY UPLC System (Waters, Milford, MA, USA) and detected using time of flight MS with an electrospray ion (ESI) source (Waters). The detection limit of the method was 0.03 g/L. The mean intra-day CV of duplicate quality control samples was 2.50%, and the inter-day CV was 9.74%.

### 2.5. Blood Sample Collection and Determination of Serum Albumin and Ferritin

Fasting blood samples (5 mL) were collected with vacutainer blood collection tubes (BD Biosciences, Franklin Lakes, NJ, USA) from lactating women (30–330 days postpartum) in the morning. Serum albumin and ferritin were measured by an automated biochemical analyzer (Hitachi 7600, Tokyo, Japan) and radioimmunoassay (Northern Institute of Biotechnology, Beijing, China), respectively.

### 2.6. Statistical Analysis

Descriptive statistics were computed for both continuous and categorical variables. All continuous variables (e.g., Lf concentration, maternal age, maternal BMI) were tested for normality. The mean and standard deviation were used for those symmetric variables. Median and quartiles were used for skewed variables. ANOVA was used for comparisons of the mean Lf concentration with three or more groups including lactation stages, geographical regions and ethnicities. After controlling for the lactation stage, the Tukey method was used for adjusting multiple comparisons when overall effects were statistical significant. The general linear model was used to examine the relationship between milk Lf concentration and factors including lactation stages, geographical regions, ethnicities, milk volume, maternal age, maternal BMI, maternal socioeconomic status, iron status, dietary intake, et al. Only those factors with a *p*-value less than 0.2 were selected to enter the multivariate model. A significance level (α) of 0.05 was used in the final models. All analyses were conducted with SAS 9.4 release (SAS Inc., Cary, NC, USA) or GraphPad Prism software (GraphPad, San Diego, CA, USA).

## 3. Results

### 3.1. Characteristics of Subjects and Samples

General information of subjects and samples is listed in Table 1. Mean maternal age of all subjects was 26.6 years, and mean BMI of all mothers at different lactation stages was 23.0 kg/m^2^. Mean birth weights of term and preterm infants were 3322 g and 3206 g, respectively. The mean weight for age Z-score was −0.11% and 51.4% of the studied infants were boys. Two weeks prior to the milk collection, the prevalence of diarrhea and respiratory disease in infants was 7.07% and 5.35%, respectively. Of the studied infants, 5.2% were delivered preterm (less than 37 gestational weeks). Colostrum, transitional milk, and mature milk were defined as the milk within 0–7 days, 8–14 days, and more than 14 days postpartum, respectively. About one quarter of the milk samples were colostrum and transitional milk, respectively, whereas approximately half of the sub-sample was mature milk. Cesarean section rate was 44.5%, primiparous rate was 76.1%, and parity was mainly 1 or 2 (Table 1). Half of the subjects lived in urban regions.

### 3.2. Milk Lf Concentrations during Lactation

**Figure 1:**
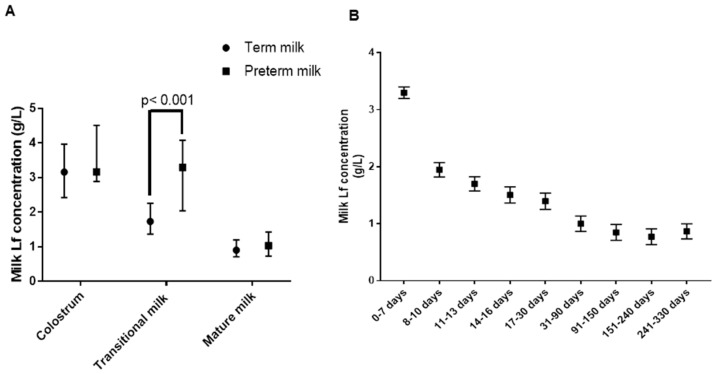
Milk Lf concentrations change among various lactation stages in Chinese populations. (**A**) Concentrations of Lf in term and preterm milk from three lactation stages. Data are representative of median (P25, P75). *n* = 198, 218, 365 for term colostrum, transitional, and mature milk, and *n* = 9, 8, 26 for preterm colostrum, transitional, and mature milk; overall *p* < 0.001; colostrum vs. transitional milk, colostrum vs. mature milk, transitional milk vs. mature milk, all *p* < 0.001, ANOVA with the Tukey adjusted method was used. (**B**) Milk Lf concentrations during the whole course of lactation. 0–7 days (*n* = 207), 8–10 days (*n* = 99), 11–13 days (*n* = 95), 14–16 days (*n* = 72), 17–30 days (*n* = 68), 31–90 days (*n* = 72), 91–150 days (*n* = 72), 151–240 days (*n* = 67), and 241–330 days (*n* = 72). Data are representative of means ± SD.

Median Lf concentration in milk obtained from mothers who delivered at term was 3.16 g/L, 1.73 g/L and 0.90 g/L for colostrum, transitional milk, and mature milk, respectively. Median Lf concentration in milk obtained from mothers who delivered preterm was 3.16 g/L, 3.29 g/L and 1.03 g/L for colostrum, transitional milk, and mature milk, respectively. As shown in Figure 1A, Lf concentrations at the three stages differed significantly (overall *p* < 0.001, colostrum vs. transitional milk, colostrum vs. mature milk, and transitional milk vs. mature milk, all *p* < 0.001). A similar difference was seen for preterm milk, but the concentration of Lf was higher in transitional preterm milk than in transitional term milk (Figure 1A). Preterm milk had a trend of 0.31 g/L higher Lf concentration than full-term milk (*p* = 0.06) during the entire course of lactation. After stratification by stage of lactation, only the mean Lf concentration of transitional milk was significantly greater in preterm milk than in full-term milk (3.13 g/L vs. 1.85 g/L, *p* < 0.001). The Lf concentration of colostrum was greatest, declined significantly during the first month of lactation (*p* < 0.05) and reached a plateau after that (Figure 1B).

### 3.3. Maternal Factors and Milk Lf Concentration

**Figure 2:**
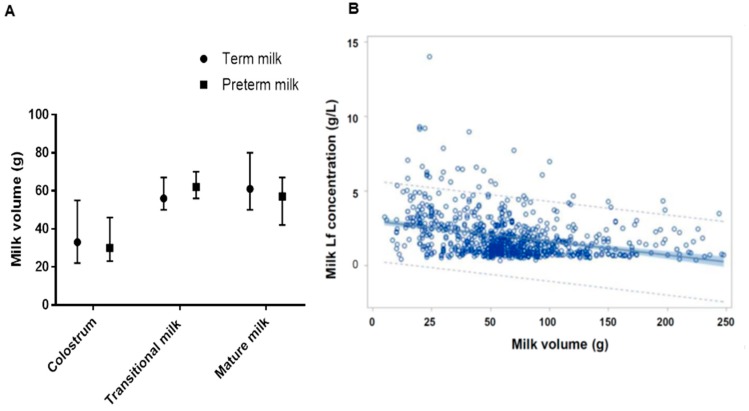
The amount of milk expressed from one breast and the relationship with milk Lf concentration. (**A**) The amount of milk expressed from one breast, which is shown as median (P25, P75). (**B**) Relationship between the amount of milk expressed from one breast and milk Lf concentration.

After controlling for geographic area and ethnicity, various maternal factors that might potentially affect Lf concentrations were analyzed. The expressed volume of term and preterm milk from one breast was similar at each stage of lactation (Figure 2A). Lf concentration was negatively associated with the expressed volume of milk; for every 50 g increase in the amount of milk expressed from one breast, the Lf concentration decreased by 0.15 g/L (*p* = 0.014) (Figure 2B). Lf concentrations at 0–7 days, 8–10 days, or 11–13 days were significantly greater than the one after 31 days. Maternal BMI (*p* = 0.36), age (*p* = 0.28), mode of delivery (*p* = 0.27), and parity (*p* = 0.16) were not significantly correlated with milk Lf concentration. Amounts of milk, meat, or soy source protein intake were not significantly correlated with milk Lf concentration. Neither was maternal serum albumin concentration (46.2 ± 2.7 g/L) significantly correlated with the milk Lf concentration (*p* = 0.94), suggesting that nutritional status of mothers as assessed by these biomarkers did not affect milk Lf concentration. However, maternal serum ferritin concentration (median (P25, P75): 19.3 μg/L (8.9 μg/L, 38 μg/L)) was negatively associated with milk Lf concentration (beta = −0.19 mg/100 g, *n* = 206, *p* = 0.047), indicating that milk Lf concentration may be associated with maternal iron status.

### 3.4. Milk Lf Concentration among Geographical Regions and Ethnicities in China

**Figure 3:**
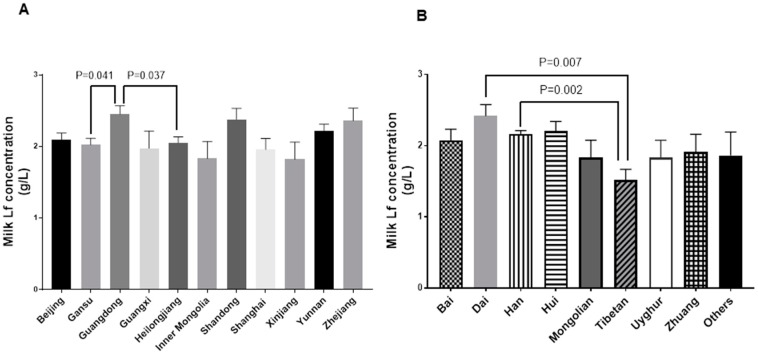
Milk Lf concentrations among various geographical regions and ethnicities in China (**A**) Average milk Lf concentrations from three lactation stages in 11 regions, overall *p* = 0.02, Guangdong vs. Heilongjiang, *p* = 0.037; Guangdong vs. Gansu *p* = 0.041. (**B**) Average milk Lf concentrations in different Chinese populations, overall *p* = 0.01, Dai vs. Tibetan, *p* = 0.007; Han vs. Tibetan, *p* = 0.002. General linear model with Tukey adjusted method was used. Data are shown as means ± SD.

After controlling for stage of lactation time and volume of breast milk expressed, the Lf concentration was significantly greater in milk from Guangdong mothers than in milk from Heilongjiang and Gansu mothers (overall *p* = 0.02, Guangdong (1.91 g/L) vs. Heilongjiang (1.44 g/L), *p* = 0.037; Guangdong (1.91 g/L) vs. Gansu (1.43 g/L) *p* = 0.041; Figure 3A). Further, after controlling for the stage of lactation and volume of breast milk pumped out, Lf concentration was significantly greater in milk from mothers of Dai and Han ethnicity mothers than in milk from mothers of Tibetan ethnicity (overall *p* = 0.01, Dai (1.80 g/L) vs. Tibetan (0.99 g/L), *p* = 0.007; Han (1.62 g/L) vs. Tibetan (0.99 g/L), *p* = 0.002, Figure 3B).

## 4. Discussion

Our study further supports that milk Lf concentrations dynamically change during lactation. The finding that milk Lf concentration is highest in colostrum, decreases considerably during the first month, and then remains relatively stable, is consistent with previously reported changes in milk Lf throughout lactation in different geographical areas [13]. This change may reflect various biological functions of milk Lf during different stages of infant development. One of the biggest challenges neonates face is the transition from a clean uterine environment to an environment filled with a variety of pathogens [16]. Lf is known to play important roles in intestinal development and immunity, thus promoting resistance to infection and more rapid growth. *In vitro* and *in vivo* studies conducted in human infants and animal models have revealed that Lf is partly resistant to proteolytic digestion [17,18,19,20]. Additionally, some Lf peptides generated from incomplete digestion show more potent effects than the intact form [21]. Intact Lf and Lf peptides exert multiple effects on the intestine by either binding to Lf receptors on the cell membrane or binding to gene promoters in the nucleus of intestinal epithelial cells [22]. Following uptake by intestinal epithelial cells, exogenous Lf can be transported through the enterocyte and enter the systemic circulation [23,24,25]. High concentrations of Lf in milk from early lactation may relate to its beneficial effects on development of immunity. Clinical trials have demonstrated protective effects of bovine Lf supplements against neonatal sepsis in preterm infants [7,26]. The high concentration of undigested Lf and Lf peptides in early life may stimulate intestinal proliferation, whereas a lower concentration of Lf in late lactation may promote intestinal differentiation [27].

A higher concentration of milk Lf was found in preterm transitional milk than in term transitional milk, whereas concentrations of Lf were similar in colostrum and mature milk from mothers of preterm and term infants. This result suggests that premature neonates may benefit from a higher concentration of milk Lf for a longer period of time. In two recent publications, the highest concentration of Lf was found in extremely preterm milk (<28 weeks of gestation or 24–27.6 week of gestational age) [28,29] compared with milk from mothers of preterm infants of later gestational age (28–36 weeks of gestation) and milk from mothers of term infants (37–41 weeks of gestation), suggesting that gestational age influences milk Lf concentration. Although only moderately preterm milk was collected for the current study, a significant difference was observed for transitional milk. Since, the sample size was not large (*n* = 8), more samples should be studied to confirm our findings.

Several maternal factors significantly corrected with the Lf concentration in human milk. A previous review of factors that affect the concentration of Lf in breast milk revealed conflicting reports of the relationship of maternal factors to breast milk Lf [30]. The present study showed that maternal age, maternal BMI, mode of delivery, parity, maternal serum albumin level, and protein intake were not significantly correlated with milk Lf concentration, whereas the amount of milk expressed from one breast and maternal serum ferritin were negatively correlated with milk Lf concentration. Maternal BMI, serum albumin concentration, and protein intake indicate nutritional status of the subjects, but appear to be unrelated to milk Lf concentration, as has been reported in previous studies [31,32]. Milk Lf concentration was negatively correlated with the amount of milk expressed from one breast, and this dilution effect was seen in previous studies [28,33]. Serum ferritin is an indicator of iron status and is positively correlated with the size of total body iron stores [34]. Although the iron saturation of Lf in human milk is only around 10% [35], the high concentration of milk Lf and high affinity of Lf to iron may have led to the decrease in maternal serum ferritin 30–330 days postpartum in the current study. According to two previous studies conducted in other countries, maternal iron status was reported to not be correlated with milk Lf concentration [36,37]. In the Peruvian study [37], iron and Lf concentrations, measured at postpartum days 2 and 30, did not significantly differ between anemic and non-anemic mothers. In the Indian study [36], concentrations of hemoglobin, serum iron and ferritin in anemic and non-anemic lactating mothers on day 1, at 14 weeks, and at 6 months postpartum were not correlated with milk Lf concentration. To our knowledge, a negative association between milk Lf and maternal iron status has not been reported previously.

Milk Lf concentration was found to be associated with geographical region and ethnicity. Milk from Guangdong mothers contained a higher concentration of Lf than did milk from Gansu and Heilongjiang mothers. Guangdong, Gansu, and Heilongjiang are located in Southern, Northwestern, and Northeastern China, respectively. The climate differs considerably between Guangdong and the other two areas. Guangdong has a short and mild winter, whereas Gansu and Heilongjiang have a long and cold winter. The annual mean temperature is 21.8 °C, 9.3 °C, and 8 °C in Guangdong, Gansu, and Heilongjiang, respectively. Due to a booming economy and high demand for labor in Guangdong, a high percentage of its residents are migrants from other provinces in China. Hence, regional genetic variation may not be the major cause of differences in milk Lf between Guangdong and Gansu or Heilongjiang. Instead, climate and factors associated with it may affect milk Lf. Ethnicity was correlated with milk Lf concentration as well. Milk from Dai and Han mothers contained a higher concentration of Lf than did milk from Tibetan mothers. There are 56 diverse populations in China, and genetic variations exist. Han Chinese constitute more than 90% of China’s population. Dai is a major Chinese ethnic minority group residing in rural areas of the southern part of Yunnan, a southwestern province in China. Tibetans live on the Tibetan Plateau, which is characterized by high altitude, low oxygen levels, and high radiation [38]. To adjust to extreme living conditions, Tibetans developed unique genetic variants during the past 25,000 years of residency [39,40]. Based on genome-wide SNP analysis, the genetics of Tibetans differ significantly from Han and Dai populations [39]. Possibly Lf gene variants in Tibetans cause their lower milk Lf concentration. 

UPLC/MS was used to determine milk Lf concentration in the present study. UPLC/MS is a highly sensitive, accurate, selective, and efficient method to quantitate proteins and metabolites [41,42] and it has been established [15,43] and validated using a signature tryptic peptide for human milk Lf [15]. A variety of other methods has been applied to measure Lf concentrations in milk, such as ELISA, radial immunodiffusion, immunoelectrophoresis, and SDS-PAGE [13], but results generated by different groups/studies exhibit high variations and are difficult to compare since different methods were used. Among the listed methods, ELISA, an immunological method, is most widely used. Although several studies have used ELISA to quantify Lf in milk, wide variations still exist probably because multiple factors markedly influence the results, such as antibody specificity and the purity of the Lf standard.

A limitation of the current study is its cross-sectional study design, which may not provide a causal relationship between the factors explored that were significantly associated with milk Lf concentration. A strength is the large sample size, which may have led to fewer false negative results.

To our knowledge, this is the largest-scale study yet conducted on factors affecting human milk Lf concentrations. In the present study, we report for the first time how milk Lf concentrations vary throughout the course of lactation in different Chinse populations. Milk Lf concentrations dynamically change throughout lactation, transitional preterm milk contains a higher concentration of milk Lf than does transitional term milk, and the amount of milk expressed from one breast, maternal serum ferritin, geographic region, and ethnicity are associated with milk Lf concentration.

## Figures and Tables

**Table 1:** Characteristics of study subjects.

Characteristics	Mean ± SD or Proportion (%) or Median (P25, P75)
Maternal age (year)	26.6 ± 4.2 (*n* = 824)
Maternal BMI (kg/m^2^)	23.0 ± 3.5 (*n* = 824)
Birth weight of term infants (g)	3322 ± 433 (*n* = 781)
Birth weight of preterm infants (g)	3026 ± 604 (*n* = 43)
Weight-for-age Z score	−0.11 (−2.31, 2.09)
Infant’s gender (boy)	51.40%
Preterm	5.2% (*n* = 43)
Lactation stage	
Colostrum	25.1% (*n* = 207)
Transitional milk	27.4% (*n* = 226)
Mature milk	47.5% (*n* = 391)
C-section rate	44.50%
Primiparous rate	76.10%
Residential area	
Urban	50%
Rural region	50%
Diarrhea during the previous two weeks	7.07%
Respiratory diseases during the previous two weeks	5.35%
